# Psychological Symptoms in Health Professionals in Spain After the First Wave of the COVID-19 Pandemic

**DOI:** 10.3389/fpsyg.2020.606121

**Published:** 2020-12-18

**Authors:** María Dosil, Naiara Ozamiz-Etxebarria, Iratxe Redondo, Maitane Picaza, Joana Jaureguizar

**Affiliations:** ^1^Department of Research and Diagnostic Methods in Education, Universidad del País Vasco/Euskal Herriko Unibertsitatea UPV/EHU, University of the Basque Country UPV/EHU, Leioa, Spain; ^2^Department of Developmental and Educational Psychology, Universidad del País Vasco/Euskal Herriko Unibertsitatea UPV/EHU, University of the Basque Country UPV/EHU, Leioa, Spain; ^3^Department of Didactics and School Organization, Universidad del País Vasco/Euskal Herriko Unibertsitatea UPV/EHU, University of the Basque Country UPV/EHU, Leioa, Spain

**Keywords:** healthcare professionals, stress, anxiety, depression, post-traumatic stress, compassion fatigue, COVID-19

## Abstract

Following the declaration of the COVID-19 outbreak as a global pandemic in March 2020, a state of alarm was decreed in Spain. In this situation, healthcare workers experienced high levels of stress, anxiety and depression due to the heavy workload and working conditions. Although Spain experienced a progressive decline in the number of COVID-19 cases until the last week of May (when a flattening of the case curve was achieved) and the work overload among health workers was substantially reduced, several studies have shown that this work overload is associated with the later emergence of psychological symptoms induced by stress. The aim of the present study was to evaluate the levels of stress, anxiety, depression, post-traumatic stress and compassionate fatigue in health professionals. The sample consisted of 973 health professionals 16.5% men, 82.9% women, and one non-binary person. The data were collected through an online questionnaire sent to the participants by e-mail. DASS-21 was used to measure anxiety, stress and depression, PCL-C to measure post-traumatic stress and ProQOL -vIV to measure compassion fatigue. In addition, other descriptive variables that could be related to these levels of psychological symptomatology were evaluated. The results reveal that after the work overload experienced during the COVID-19 pandemic, healthcare workers report psychological symptoms, post-traumatic stress and compassion fatigue. It is therefore recommended that these professionals be provided with psychological help in order to reduce the emotional impact of COVID-19, and consequently improve their mental health.

## Introduction

At the end of December 2019, the Chinese city of Wuhan reported a novel pneumonia caused by coronavirus disease 2019 (COVID-19) (Lai et al., 2020). The subsequent outbreak of COVID-19 not only caused great public concern, but also brought about huge psychological distress, particularly for the medical staff (Cheng and Li Ping Wah-Pun Sin, 2020; García-Iglesias et al., 2020; Zhang et al., 2020). The growing number of confirmed and suspected cases, overwhelming workload, extensive media coverage, depletion of personal protective equipment, lack of specific medications and perceived inadequate support has contributed to the significant mental burden that has been carried by these health professionals (Lee et al., 2007; Lai et al., 2020; Pfefferbaum and North, 2020).

Stress reaction symptoms such as anxiety, depression, somatization and hostility have been reported during and after the previous pandemics (Mak et al., 2009). More recently, during the COVID-19 pandemic, the prevalence of depression, anxiety and stress-related symptoms were found to be 50.7, 44.7, and 73.4%, respectively, among Chinese healthcare workers (Lai et al., 2020). Likewise, another study in Turkey confirmed that 64.7% of physicians had depressive symptoms, 51.6% suffered from anxiety and 41.2% experienced stress-related symptoms in the early period of the COVID-19 outbreak (Elbay et al., 2020). Nevertheless, for the time being, very few studies have been carried out on the subject in the European context. One of these, that aimed to investigate the psychological health of Italian healthcare professionals, revealed that approximately 33.5% of them met the threshold for psychiatric morbidity. Furthermore, participants perceived their current psychological health to be worse during the COVID-19 emergency outbreak as compared to before the outbreak (Bettinsoli et al., 2020). In Spain, a study conducted with medical staff in the same time frame, reported that 46.7% of health professionals indicated suffering from stress, 37% from anxiety, 27.4% from depression and 28.9% from sleep problems, with higher levels of symptoms among women and older professionals. Furthermore, factors such as having been in contact with the virus or experiencing fear at work, triggered greater symptomatology (Dosil et al., 2020). More recently, a systematic review including 13 studies detected medium-high levels of anxiety (26.5–44.6%), depression (8.1–25%), concern and insomnia (23.6–38%) among these professionals, and found that mental health and mental functions were especially compromised on those professionals hting on the front line of battle against the virus (García-Iglesias et al., 2020).

Other important factors that have been scarcely investigated in relation with the COVID-19 are compassion satisfaction, compassion fatigue and post-traumatic stress. The little research existing on this subject has been conducted within the context of other pandemics and some previous emergency situations.

Compassion satisfaction (CS) and compassion fatigue (CF) are considered to be part of professional quality of life (ProQOL), understood as “the quality one feels in relation to their work as a helper.” While CS includes positive aspects such as perceiving that helping is in itself a worthwhile endeavor, CF is defined as “the emotional residue resulting from exposure to work with those who suffer the consequences of traumatic events” (Acinas, 2012, p. 3). Individuals who experience CF describe a feeling of tiredness or mental exhaustion that causes a general decrease in their desire, ability or energy to help other individuals (OHIO Nurses Association, 2011; Cocker and Joss, 2016).

The literature has clearly established that CF is high among all health professionals, but particularly for those who work in environments where they are confronted daily with large numbers of people for whom the outcome is potentially dire, such as in the case of those diagnosed with COVID-19 that require admission to emergency or intensive care units (Wallace et al., 2020). In fact, frequently seeing or experiencing the death and suffering of patients, or having the responsibility for deciding how to ration or use health resources, increases the risk of developing CF and moral injury among healthcare professionals during pandemics (Doherty and Hauser, 2019).

With this regard, some authors warn that healthcare providers such as critical care nurses may be particularly affected by severe emotional distress, which has been associated with the development of CF and/or burnout (Alharbi et al., 2020; Denison and Baptiste, 2020). For example, a recent study conducted by Arribas-García et al. (2020) with oncology nursing staff, reported that 41.8% of them showed moderate levels of CF. Therefore, Li et al. (2020), caution against ignoring vicarious traumatization caused by the COVID-19 pandemic, and some authors recommend close monitoring of physical and emotional wellbeing and providing education to professionals in order to reduce CF (Alharbi et al., 2019). However, all of these issues have received relatively little attention in the context of this pandemic.

Further, post-traumatic stress disorder (PTSD) is understood as a state of psychological unbalance following exposure to exceptionally threatening or horrifying events and it is characterized by a typical symptom pattern of intrusions, persistence of trauma, avoidance of relevant stimuli, emotional numbing, and physiological hyper-arousal (Deja et al., 2006). Nevertheless, subsequent empirical studies have consistently demonstrated that substantial rates of subclinical post-traumatic stress symptoms (PTSS) exist and are more persistent (Yin et al., 2020).

Many previous studies have shown that professionals such as emergency rescuers are likely to suffer from PTSD after participating in an emergency (Ozen and Sir, 2004; Wang Y. X. et al., 2020). In the context of epidemics, PTSD is also very likely to appear. For example, during the SARS epidemic of 2003, the rate of PTSD among frontline medical staff was high, with reports of up to 25.8% (Xu et al., 2004), whilst another study revealed that approximately 20% of the participants were diagnosed with PTSD 2 months after the epidemic outbreak (Chan and Huak, 2004). In fact, some studies have shown that healthcare workers are subject to early onset PTSD not at the moment, but after spending a long period of time in a horrific situation (Lazarus, 2014; Brondolo et al., 2017).

In a more recent investigation carried out in the context of the COVID-19 pandemic and involving 371 Chinese healthcare professionals, the total prevalence of post-traumatic stress symptoms was 3.8% and prevalence reached 8.8% on those subjects with high-level exposure to COVID-19 (Yin et al., 2020). However, data from European population on PTSS seems to be even higher. Hence, a Greek study conducted in April, found that criteria for a probable post-traumatic stress disorder diagnosis were met by a total of 16.7% of healthcare professionals in their sample (21.7% of women; 5.1% of men) (Blekas et al., 2020).

Finally, it is important to point out that when analyzing distress levels of these professionals, some socio-demographic variables (age, sex, professional category, etc.) or some others, such as direct exposure to COVID-19, may act as risk or protective factors. With this regard, Babore et al. (2020) found that female gender was a risk factor for that, but not the economic status, while Buselli et al. (2020) reported that some symptoms were more prevalent in the frontline staff and healthcare assistants than in the second-line staff and physicians, respectively.

As it can be observed, evidence-based evaluations targeting healthcare workers and their psychological needs in the COVID-19 pandemic are relatively scarce. The few studies that exist have been carried out above all in Asian population and have mainly been focused on the times when the pandemic was very active. However, there is very little research on this issue in Spain.

Spain is one of the countries hardest hit by the health crisis caused by the COVID-19 pandemic (Ruiz-Fernández et al., 2020). In fact, a lockdown had to be enforced on March 15, 2020, when it presented 5,753 confirmed cases and 136 deaths due to COVID-19 (World Health Organization, 2020). By April 25, 2020, the country started to ease the lockdown with a gradual lifting of restrictions due to decreasing trends in confirmed cases, hospitalizations, and daily deaths (Ministerio de Sanidad, 2020). During the mentioned period, 223,791 new cases were registered, along with 23,135 deaths. The Basque Country and Navarre were among the Spanish communities that required more time than the national average (18.33 days) to reduce the daily number of deaths. Moreover, The Basque Country presented together with La Rioja and Catalonia some of the highest rates of hospital and ICU admissions (Siqueira et al., 2020).

As cases of COVID-19 showed a progressive decline until the last week of June in our country, when a flattening of the case curve was achieved and burden placed on health workers were significantly reduced. It could be assumed that the new situation could lead to a decrease in psychological symptoms among these health professionals, since they were less exposed to danger and more aware of the improvement of the situation. Even so, several studies have shown that following this work overload, psychological symptoms can still appear due to the distress experienced previously (Ozamiz-Etxebarria et al., 2020). Nevertheless, there is a dramatic gap in the current scientific literature that actually addresses this issue.

Hence, taking into consideration all the mentioned above, the aims and hypotheses of this study were:

1. To measure the levels of stress, anxiety, depression, compassion fatigue and post-traumatic stress symptoms among health professionals in Spain after the flattening of the curve of the COVID-19. We hypothesized that all those levels would be lower than those observed at the outbreak of the pandemic. When comparing and contrasting the data, special consideration will be given to a study conducted previously by the authors at the beginning of the lockdown (Dosil et al., 2020).
2. To study the possible differences in the level of these symptoms displayed by the health professionals according to other relevant factors (such as age, gender, professional category, contact with COVID-19 and perception of social compliance of the health measures). It was hypothesized that symptoms would be greater among women, older professionals and those with greater contact with the COVID-19, and lower among nurses/auxiliaries/technicians and those who perceive that the health measures were being complied.

## Materials and Methods

### Participants

This study was carried out with a total sample of 973 health professionals: 832 (85.5%) from the Basque Autonomous Community, 14(1.4%) from Navarra, and 127(13.1%) from other communities of Spain. The participants were working professionals from various hospital centers from both the public and private sectors. Of the participants, 165 (16.5%) were men, 807 (82.9%) were women and one person was considered non-binary. With regard to age, 42 (4.3%) were aged between 18 and 25 years, 221 between 26 and 35 (22.7%), 503 (51.7%) between 36 and 55 and 207 (21.3%) over 56. Of the participants, 433(44.5%) were doctors, 318 (32.6%) were nurses, and 222 (22.9%) were auxiliaries/technicians.

### Measures and Instruments

An *ad hoc* instrument was used to collect information about whether they had had contact with any person diagnosed with COVID-19 (yes/no), and about their perception of whether people were respecting health measures (yes/no).

The *Depression and Stress Anxiety Scale-21* (DASS-21, Ruiz et al., 2017) was administered to measure stress, anxiety and depression symptoms. The DASS-21 scale is composed of 21 Likert-type items ranging from (0 = It didn’t happen to me) to (3 = It happened to me a lot, or most of the time) and are organized into 3 subscales of 7 items each: Depression, Anxiety and Stress. The total scores of each subscale is within the range of 0–21. In addition, cut-off points analyzed by Antony et al. (1998) can be used in order to categorize depressive, anxiety, and stress symptoms into the following categories: no symptoms, mild, moderate, severe, and extremely severe. The DASS-21 has shown acceptable reliability and good validity (Antúnez and Vinet, 2012). Regarding the reliability in our study, the total Cronbach’s alpha coefficient was = 0.88 for the depression scale = 0.87 for the anxiety scale = 0.82 and for the stress scale = 0.87.

Post-traumatic stress was measured using the *Post-traumatic stress scale* (*PCL-C scale*, Weathers et al., 1991, the Spanish version of Miles et al., 2008) which is a standardized self-report rating scale for PTSD that includes 17 items corresponding to the key symptoms of PTSD. The *PCL-C* is a 17-item self-rated questionnaire that is generally applied to any traumatic event. It includes a five-point Likert scale ranging from 1 (none) to 5 (extremely) for each item. The PCL-C provides a continuous score based on the number and severity of PTSD symptoms according to DSM-IV criteria. The questionnaire gives a total score, as well as allowing for the gradation of symptoms related to a stressful experience in the past according to three subscales: re-experimentation, avoidance/numbness, and hyperactivation. The higher the score, the more severe the symptoms of stress disorder. PCL-C is often used to evaluate the effects of diagnosis, intervention and treatment of post-traumatic stress disorder. It has good reliability and validity and is one of the most widely used tools in this field (Wu and Wei, 2020). Cronbach’s alpha was = 0.94.

The *Professional Quality of Life Scale* (ProQOL v. IV) is used with health professionals who are exposed to situations of trauma and suffering (Stamm, 2005). The Spanish version of ProQOL v. IV (Morante-Benadero et al., 2005) is a self-administered questionnaire consisting of 30 items rated on a 5-point Likert scale (ranging from 1 = “never” to 5 = “very often”). The ProQOL measures two main dimensions: Compassion Satisfaction (CS) (10 items) and Compassion Fatigue (CF), which is composed of two subsets of symptoms: Burnout (BO) (10 items) and secondary traumatic stress (STS) (10 items). Compassion satisfaction (CS) is the satisfaction experienced by health professionals in doing their job properly, which also includes satisfaction in the relationship with their colleagues and the feeling that the work they do is of social value (Roney and Acri, 2018). BO is a syndrome of emotional exhaustion, depersonalization and lack of personal fulfilment in the workplace, characteristics that develop as a result of continuous exposure to occupational stressors (Lim et al., 2019). Secondary traumatic stress (STS) is a set of natural emotions and behaviors that arise after learning about a traumatic event in detail, experienced by someone significant. The STS is a gradual process that does not appear as an immediate response at the first contact with the person or their history of pain. It is, rather, the cumulative effect of systematic contact with people who are experiencing a very difficult emotional situation (Morales et al., 2016). Higher scores on each of these scales are taken to indicate higher CS and CF (including BO and STS) values. The mean score is 13 for the CF subscale, 37 for the CS subscale, and 22 for the BO subscale. Stamm (Sacco et al., 2015) reported Cronbach’s alpha values of 0.80 for CF, 0.89 for CS, and 0.71 for BO, respectively (Ruiz-Fernández et al., 2020). For this study Cronbach’s alpha was CS = 0.87, BO = 0.70 and STS = 0.84.

### Procedure

The sample was recruited through non-probabilistic sampling. An online questionnaire was first created in Google Forms and sent to platforms, and through the institutional mail of the researchers. The questionnaire explains both the objectives of the study and the procedures to be followed during the questionnaire, as well as the right to voluntary withdraw from the study if appropriate. The study was approved by the Ethics Committee of the University of the Basque Country (UPV/EHU) (code M10/2020/070). For the collection of data, all the canons established by the Organic Law 15/99 on Personal Data Protection were followed. In the questionnaires, they participants were informed of the voluntary nature of their participation and of their necessary commitment to start the test. Therefore, the procedure followed is approved by the Ethics Committee and was carried out in accordance with the Helsinki Declaration of the World Medical Association.

### Data Analysis

The data were analyzed with the statistical program SPSS v.26 (Armonk, NY, United States). First, the assumptions of normality and homocedasticity of variances were checked in order to decide whether to use parametric or non-parametric tests. Specifically, the critical level of *p* < 0.05 of the Kolmogorov-Smirnov statistics was analyzed, as well as the levels of asymmetry and kurtosis. From these analyses it was concluded that the data followed a normal distribution, so the authors decided to use parametric tests.

The different levels of depression, anxiety and stress were categorized with cut-off scores proposed by Antony et al. (1998): mild, moderate, severe and extremely severe.

First, both the frequencies and the percentages of the different levels of each scale were described. Then, comparative analyses were carried out, with the t-student test, using total scores of depression, anxiety, stress, post-traumatic stress and professional quality of life as dependent variables, and as independent variables sex (woman/man), having been in contact with COVID-19 (yes/no) and if they perceived that people respected the health norms to prevent the COVID-19 (yes/no). In these cases, the interval coefficients and effect sizes are provided (Cohen, 1988). Likewise, to explore the difference in means according to variables with more than two categories (such as age and professions of the participants), ANOVAs were carried out. In this case, Bonferroni’s *post hoc* test was used to observe the differences between more than two groups.

## Results

### Levels of Anxiety, Depression, Stress, Post-traumatic Stress, and Compassion Satisfaction/Compassion Fatigue in the Study Sample

The results revealed higher percentages of extremely severe or severe levels of anxiety and stress than of depression. Furthermore, moderate levels of depression, anxiety and stress (with percentages close to 20%) can be observed (see Table 1).

**TABLE 1:** Frequencies and percentages of the perceived level of depression, anxiety and stress symptoms (none, mild, moderate, severe, and extremely severe) suffered by health professionals.

	**None**	**Mild**	**Moderate**	**Severe**	**Extremely severe**
Depression	544 (55.9%)	138 (14.2%)	181 (18.6%)	63 (6.5%)	47 (4.8%)
Anxiety	459 (47.2%)	80 (8.2%)	218 (22.4%)	101 (10.4%)	115 (11.8%)
Stress	459 (47.2%)	84 (8.6%)	209 (21.5%)	162 (16.6%)	59 (6.1%)

Post-traumatic stress levels in the sample were high (26.4%) and medium (44.7%). In contrast, the levels of Secondary Traumatic Stress (STS) were lower: 0.2% high and 19.2% medium. Burnout (BO) levels were generally medium (90.6%), while Compassion Satisfaction (CS) was high (33.2%) or medium (63.1%) (see Table 2).

**TABLE 2:** Frequencies and percentages of the perceived level of post-traumatic stress and professional quality of life symptoms (low, medium, and high) suffered by health professionals.

	**Low**	**Medium**	**High**
Post-traumatic stress	281 (28.9%)	36 (3.7%)	72 (7.4%)
CS	435 (44.7%)	614 (63.1%)	882 (90.6%)
BO	257 (26.4%)	323 (33.2%)	19 (2%)
STS	783 (80.5%)	783 (80.5%)	783 (80.5%)

*CS, Compassion Satisfaction; BO, Burnout; STS, Secondary Traumatic Stress.*

### Differences Between the Symptoms According to Gender and Age

Statistically significant differences were found according to gender for all the variables under study, with women showing higher levels than men in all cases, with medium or low effect sizes (see Table 3).

**TABLE 3:** Differences in the means of the variables according to the gender of the participants.

**Dimensions**	**Gender**	***n***	***M***	***SD***	***t***	***p***	***d*_cohen_**
Depression	Women	807	5.15	4.27	4.84	0.001***	0.43
	Men	165	3.41	3.88			
Anxiety	Women	807	5.10	4.13	9.60	0.001***	0.72
	Men	165	2.56	2.84			
Stress	Women	807	9.55	4.39	7.48	0.001***	0.63
	Men	165	6.74	4.49			
Post-traumatic stress	Women	807	35.88	13.2	6.66	0.001***	0.55
	Men	165	29.11	11.6			
CS	Women	807	37.99	6.96	2.40	0.017*	0.21
	Men	165	36.58	6.65			
BO	Women	807	29.89	5.29	2.09	0.037*	0.18
	Men	165	28.95	4.92			
STS	Women	807	17.06	7.22	3.28	0.001**	0.29
	Men	165	15.06	6.67			

CS, Compassion Satisfaction; BO, Burnout; STS, Secondary Traumatic Stress. *p < 0.05; **p < 0.01; ***p < *0.001.*

In terms of age differences, the results of the ANOVA revealed that participants aged 26–35 years scored higher on depression, anxiety, stress and post-traumatic stress. The oldest participants of the sample (35–55 and <56) showed more Burnout (BO) than the youngest participants, whilst the youngest (18–25 years) showed the lowest levels of Secondary Traumatic Stress (STS). And finally, the highest levels of Compassion Satisfaction (CS) were found among the 26–35, and 36–55 years-old participants (see Table 4).

**TABLE 4:** Differences in the means of the variables under study according to the age of the participants.

**Dimensions**	**Age**	***n***	***M***	***SD***	***F (gl)***	***p***	**η*2***	***Post hoc***
Depression	18–25 26–35 36–55 >56	42 221 503 207	4.81 5.74 4.71 4.28	4.50 4.28 4.14 4.34	4.68 (3)	0.003**	0.014	2–3 2–4 3–2 4–2
Anxiety	18–25 26–35 36–55 >56	42 221 503 207	5.33 5.64 4.70 3.42	4.55 3.96 4.10 3.65	11.57 (3)	0.001***	0.035	1–4 2–3 2–4 3–2 3–4 4–1 4–2 4–3
Stress	18–25 26–35 36–55 >56	42 221 503 207	8.81 10.37 9.09 7.69	4.83 4.07 4.48 4.71	12.98 (3)	0.001***	0.040	2–3 2–4 3–2 3–4 4–2 4–3
Post-traumatic Stress	18–25 26–35 36–55 >56	42 221 503 207	32.74 36.57 35.11 32.16	13.26 12.60 13.32 13.20	4.54 (3)	0.004**	0.014	2–4 3–4 4–2 4–3
CS	18–25 26–35 36–55 > 56	42 221 503 207	37.55 38.19 38.20 36.14	8.55 6.52 6.76 7.42	4.72 (3)	0.003**	0.014	2–4 3–4 4–2 4–3
BO	18–25 26–35 36–55 >56	42 221 503 207	25.67 29.31 30.10 30.01	5.58 5.31 5.18 5.05	10.11 (3)	0.001***	0.030	1–2 1–3 1–4 2–1 3–1 4–1
STS	18–25 26–35 36–55 >56	42 221 503 207	13.26 16.83 16.76 17.14	7.31 7.23 7.05 7.24	3.55 (3)	0.014*	0.011	1–2 1–3 1–4 2–1 3–1 4–1

CS, Compassion Satisfaction; BO, Burnout; STS, Secondary Traumatic Stress. *p < 0.05; **p < 0.01; ***p < *0.001.*

### Differences Between the Symptoms Studied According to Professional Category

Table 5 shows the differences in the means of the variables under study according to professional category. Significant differences are observed in all of the variables except for Secondary Trauma Stress (STS). Levels of depression, anxiety, stress and post-traumatic stress were significantly higher in nurses and technicians/auxiliaries than in physicians, with no differences found between nurses and auxiliaries, except for anxiety, with technicians/auxiliaries reporting the highest levels. Compassion Satisfaction (CS) was higher in the technicians/auxiliaries than in the nurses, whilst this was higher in the nurses than in the physicians. In contrast, Burnout (BO) was higher in doctors than in nurses, and no difference was found between nurses and technicians/auxiliaries.

**TABLE 5:** Differences in the averages of the variables under study according to professional category.

**Dimensions**	**Profession**	***n***	***M***	***SD***	***F (gl)***	***p***	**η*2***	***Post hoc***
Depression	Doctors Nurses Auxiliaries/technicians	433 318 222	4.10 5.36 5.60	3.90 4.34 4.55	12.82 (2)	0.003**	0.026	1–2 1–3 2–1 3–1
Anxiety	Doctors Nurses Auxiliaries/technicians	433 318 222	3.27 5.35 6.41	3.18 4.12 4.54	56.69 (2)	0.001***	0.011	1–2 1–3 2–1 2–3 3–1 3–2
Stress	Doctors Nurses Auxiliaries/technicians	433 318 222	8.40 9.49 9.77	4.60 4.28 4.61	8.91 (2)	0.001***	0.018	1–2 1–3 2–1
Post–traumatic Stress	Doctors Nurses Auxiliaries/technicians	433 318 222	31.72 36.54 36.16	11.73 14.21 13.26	21.73 (2)	0.001***	0.043	1–2 1–3 2–1 3–1
CS	Doctors Nurses Auxiliaries/technicians	433 318 222	36.16 38.19 40.11	6.79 6.98 6.57	25.88 (2)	0.001***	0.051	1–2 1–3 2–1 2–3 3–1 3–2
BO	Doctors Nurses Auxiliaries/technicians	433 318 222	30.19 29.09 29.66	5.37 5.21 5.07	4.05 (2)	0.018*	0.008	1–2 2–1
STS	Doctors Nurses Auxiliaries/technicians	433 318 222	16.78 16.54 16.80	7.04 7.39 7.12	0.123 (2)	0.885	0.001	–

CS, Compassion Satisfaction; BO, Burnout; STS, Secondary Traumatic Stress. *p < 0.05; **p < 0.01; ***p < 0.001.

### Differences in Symptomatology of the Participants Depending on Variables Associated With the COVID-19 Pandemic

We analyzed whether there were statistically significant differences in the variables under study between those who had been in direct contact with COVID-19 and those who had not. As can be observed in Table 6, those who had been in direct contact with the virus had higher levels of depression, anxiety, stress and post-traumatic stress, although no statistically significant differences were found in Compassion Satisfaction (CS), Burnout (BO), and Secondary Traumatic Stress (STS).

**TABLE 6:** Results of univariate analysis of variance for different symptoms according to whether the participants had been in contact with COVID-19.

**Dimensions**	**Contact with COVID-19**	***n***	***M***	***SD***	***t***	***p***	***d*_cohen_**
Depression	Yes	829	5.04	4.30	3.58	0.001***	0.31
	No	144	3.79	3.76			
Anxiety	Yes	829	4.87	4.11	3.91	0.001***	0.37
	No	144	3.45	3.52			
Stress	Yes	829	9.30	4.48	3.75	0.001***	0.34
	No	144	7.77	4.66			
Post-traumatic Stress	Yes	829	35.43	13.27	4.15	0.001***	0.39
	No	144	30.53	12.03			
CS	Yes	829	37.66	7.03	–0.705	0.428	0.06
	No	144	38.10	6.69			
BO	Yes	829	29.76	5.27	0.746	0.456	0.07
	No	144	29.40	5.31			
STS	Yes	829	16.85	7.20	1.50	0.135	0.13
	No	144	15.88	7.34			

CS, Compassion Satisfaction; BO, Burnout; STS, Secondary Traumatic Stress. ***p < *0.001.*

In relation to the professionals’ perception of society’s respect for health measures, there were statistically significant differences in the dimensions of depression, anxiety, stress and post-traumatic stress, showing a higher level of symptoms in those who indicate that health measures are not being respected (see Table 7).

**TABLE 7:** Results of univariate analysis of variance for different symptoms according to the perception of whether society is respecting health measures.

**Dimensions**	**Respect of measures**	***n***	***M***	***DT***	***t***	***p***	***d*_cohen_**
Depression	Yes	440	4.09	4.03	5.16	0.001***	0.33
	No	533	5.48	4.34			
Anxiety	Yes	440	3.79	3.71	6.28	0.001***	0.40
	No	533	5.38	4.20			
Stress	Yes	440	8.14	4.55	5.93	0.001***	0.38
	No	533	9.85	4.39			
Post-traumatic stress	Yes	440	32.21	12.45	5.50	0.001***	0.35
	No	533	36.78	13.46			
CS	Yes	440	37.43	6.43	1.21	0.233	0.08
	No	533	37.97	7.39			
BO	Yes	440	29.40	5.03	1.66	0.101	0.11
	No	533	29.96	5.45			
STS	Yes	440	16.06	6.84	2.57	0.010*	0.17
	No	533	17.24	7.40			

CS, Compassion Satisfaction; BO, Burnout; STS, Secondary Traumatic Stress. *p < *0.05*; ***p < *0.001.*

## Discussion

The present research stems from a preliminary study on the stress of healthcare professionals in the Basque Autonomous Community and Navarre (Spain) during the first months of the COVID-19 pandemic. This study prompted the conclusion that it was important to treat possible cases of post-traumatic stress caused by this pandemic (Dosil et al., 2020). Therefore, in this second study, in addition to some of the previously studied factors (depression, anxiety, stress) post-traumatic stress disorder (PTSD) and Professional quality of life (ProQOL) were added. As has been the case in other pandemics (Mak et al., 2009), in this study there are numerous cases of health professionals reporting symptoms such as depression, anxiety and stress. Against what we thought, there are more symptoms among health professionals in the current study than those found in the previous study by the same team (Dosil et al., 2020) and also higher than those studied in the systematic review conducted by García-Iglesias et al. (2020). Therefore, it appears that although the questionnaire was conducted at a time when professionals did not have as much work as at the onset of the pandemic, symptoms were already accumulating since its beginning. However, people who have symptoms of stress are still fewer than those found among health workers in China. In the study by Lai et al. (2020), 73.4% of participants reported stress symptoms, a significantly higher percentage than those found in the present and previous studies (Dosil et al., 2020). On the contrary, compared to the study in Turkey (Elbay et al., 2020), more people in the present study have levels of stress, but far fewer have levels of depression. Therefore, it seems that there are many differences of symptoms among countries and future studies should study what factors could be affecting them.

With regard to post-traumatic stress, in a study conducted with frontline health professionals working with COVID-19 in China, the average PCL-C scores were very similar to those found in the present study (M = 33.73 ± 1.556) (Wu and Wei, 2020). As already mentioned, post-traumatic stress can be developed after exposure to exceptionally threatening events and its main symptoms are re-experiencing them, being on alert and having a continuous feeling of threat (Wang Y. X. et al., 2020). As many studies have shown, a critical situation such as the COVID-19 pandemic can intensify post-traumatic stress among health workers, and this stress level is higher than in the general population (Ozen and Sir, 2004; Fjeldheim et al., 2014; Wang Y. X. et al., 2020). In the present study, 28.9% of professionals showed low levels, 3.7% medium levels and 7.4% high levels of post-traumatic stress. These percentages are higher than those found during the 2003 SARS epidemic, where 25.8% of physicians had symptoms of post-traumatic stress (Xu et al., 2004), and also higher than in another study indicating that 20% of participants were diagnosed with PTSD 2 months after the epidemic outbreak (Chan and Huak, 2004).

In the case of the COVID-19 pandemic, more participants in this study also had symptoms of post-traumatic stress than those in a study in China (Yin et al., 2020) and participants in a Greek study in April (Blekas et al., 2020). Therefore, it could be said that there are more cases of professionals with post-traumatic stress in this study than in most of the studies we have found in both the SARS and COVID-19 pandemics. These results show that Spanish healthcare professionals are experiencing greater suffering than professionals in other countries. This may be due to the fact that Spain is one of the countries most affected by the health crisis caused by the COVID-19 (Ruiz-Fernández et al., 2020).

A positive finding of this study is that a high percentage of the participants have compassion satisfaction. In fact 90.6% of them showed a high compassion satisfaction. Healthcare work is a vocational job and that is why respondents could be so satisfied. Still, although there are many participants who experienced high compassion satisfaction, we cannot ignore that there are also many healthcare providers who suffer from secondary traumatic stress (STS) that may increase by knowing in detail the characteristics of the traumatic events of the patients (Morales et al., 2016). There are also respondents in the study who report symptoms of burnout. Several studies have shown that physicians who perform high-risk procedures are at increased risk of burnout (Lacy and Chan, 2018). The COVID-19 pandemic poses a high risk to physicians, many of whom are infected, so this could be a reason for burnout.

It may seem contradictory that participants in this study report moderate and high levels of compassion satisfaction as well as a variety of psychological symptoms. However, a study conducted with health professionals working in critical incident services revealed that participants were at risk of compassion fatigue whilst also showing high potential for compassion satisfaction (Wee and Myers, 2003). This could occur due to the fact that although professionals recognize the stress level associated with their work, it also provides significant rewards that somehow outweigh the stress and mitigate exhaustion. Future studies should explore the distinctive characteristics of these individuals (personality, resilience, attitude to death, etc.) who, under the same work circumstances as those with high levels of anxiety and depression, and despite the risks, fatigue and workload, continue to show high scores in compassion satisfaction.

With regard to gender, as has been the case in most studies carried out both in the general population (Ozamiz-Etxebarria et al., 2020) and with healthcare professionals (Dosil et al., 2020), women presented higher levels than men in all symptoms. This is why in this pandemic, special attention must be paid to women, who seem to be the ones suffering most in different parts of the world.

In terms of age, as in our preliminary study, younger health workers showed higher levels of stress, anxiety, depression and post-traumatic stress. In this second study, professionals between 26 and 35 years particularly stand out, which is in accord with the findings of various other studies (Lai et al., 2020). The same pattern of results was found with STS symptomatology in the recent study, with the highest levels reported among those in the 26–35-year age range. One possible explanation for this could be that these workers, who have less experience because they are young, are more impressionable and feel more impacted by situations that are perhaps more expected and known by their older counterparts.

With regard to compassion satisfaction, in our research people within the 35–55 years age range report the highest levels. This is consistent with the results of a recent study conducted in China where being aged 36 years or older was positively associated with compassion satisfaction (Wang J. et al., 2020). This could be because in this age range professionals have more stability at work, and could enjoy more helping patients.

In relation to burnout, a rather different trend can be observed with respect to age. In this case, professionals over the age of 35 (35–55 and <56) showed the highest levels. Older workers can face more barriers and stressors at work such as physical strength limitations and health concerns, gaps related to using new technology, and engagement in work (United Nations Economic Commission for Europe, 2014), which could even be exacerbated in the specific circumstances of this pandemic. Older workers also have their own expectations of retirement age, and the closer they are, the more likely they are to disengage from work (Damman et al., 2013) or to feel overwhelmed by their workload.

In addition, the results of the current study show that stress and anxiety levels are higher in nursing professionals, particularly auxiliary technicians, although burnout is higher among doctors. Our hypothesis was that perhaps the doctors were more symptomatic since they are the professionals who make the final decisions. However, the results are in line with the findings of various studies indicating that healthcare providers such as critical care nurses are particularly affected by severe emotional distress (Alharbi et al., 2020; Denison and Baptiste, 2020). In fact, nurses and assistants have the most direct contact with patients and their families, so they are more likely to be emotionally involved, which can lead to higher levels of emotional problems, such as stress and anxiety as well as greater level of compassion satisfaction when the emotional demands are adequately addressed.

Finally, and as mentioned previously, the results reported here, as in the preliminary study, suggest that being in contact with COVID-19 is associated with higher levels of depression, anxiety and stress. Furthermore, PTSD levels are also higher in professionals who have been in contact with the virus. The presence of these symptoms is common in this situation where one may believe that he/she is vulnerable to infection, and the uncertainty of unknown infections could lead to this symptomatology (Chew et al., 2020; Dosil et al., 2020). Exactly the same pattern of symptoms is observed among those who perceive that security measures have not been respected. In fact, those who believe that society is not adequately complying with health measures, are probably afraid of new outbreaks, which may be increasing their levels of anxiety, depression, stress, post-traumatic stress levels and secondary traumatic stress. The feeling of lack of unity among the population could lead to psychological symptoms among the professionals.

Despite the interesting results found in the study, it is important to point out some limitations. First, the cross-sectional nature of the design employed here means that there was no longitudinal follow-up. It would be interesting to compare the results with others found previously or later, in order to observe the evolution of the symptoms at different moments in time with the same sample. Second, the voluntary nature of the survey may have introduced a response bias if the non-respondents were either too symptomatic to respond, or too relaxed, and therefore not interested in this survey. As for the professionals who answered the questionnaire, although they all answered at the same time, it must be taken into account that each professional could be living different life circumstances at the time of answering the questionnaire. Moreover, not all the autonomous communities have experienced the same number of infections by COVID-19 nor have they the same health resources, so the results obtained should be taken with caution. Future studies should include more basic socio-demographic data, such as marital status, housemate number or number of children, or perceived emotional/social support, that may have a role in moderating the impact of the work overload.

It is also important to mention that most of the people who have answered the questionnaire are women (82.9%). This may be due to the fact that currently the feminine gender is growing among health professionals (Ponce, 2006). In any case, this gender imbalance in the study should be considered as a limitation.

Lastly, future studies should include a control group to determine whether this symptomatology is associated with being a health professional or whether it occurs equally in the general population.

In any case, the findings of this study make a general contribution to existing knowledge regarding the psychological symptomatology of these professionals in the context of an unprecedented health emergency in the last century, and opens the door to further research in the near future.

## Conclusion

The study shows that health professionals are suffering from psychological symptoms such as stress, anxiety and depression, compassion fatigue and post-traumatic stress, even after the most difficult times of the pandemic. We have exhausted workers with fear of new outbreaks. For this reason, we recommend the implementation of psychological support (Conversano et al., 2020; Petzold et al., 2020) and timely interventions for health workers who present psychological symptoms due to the work overload suffered amid the COVID-19 crisis.

Having psychologically healthy medical staff will be helpful for preventing employment losses due to emotional suffering and will improve the quality of patient care. It means providing more resources to society and specifically to health personnel. Among these measures it is important to support the professionals by expanding the staff with more professionals and more resources. Furthermore, emphasizing the areas of direct care and attention to family members could be of great interest. Another interesting measure would be to provide training to health personnel about the pandemic and to the population in general to raise awareness and prevent contagion. In addition, professionals should have protective uniforms and adequate space to carry out their work in dignified conditions.

Urgent action must be taken to protect the mental health of health professionals, especially for those who are at the frontline of the COVID-19 pandemic. This is not only necessary to maintain a robust health system to meet this challenge, but it is also something we certainly owe to health professionals for the tremendous sacrifices they are doing.

## Data Availability Statement

The raw data supporting the conclusions of this article will be made available by the authors, without undue reservation.

## Ethics Statement

The studies involving human participants were reviewed and approved by the Comité de Ética para las Investigaciones relacionadas con Seres Humanos (CEISH) (University of the Basque Country). The participants provided their written informed consent to participate in this study.

## Author Contributions

IR, MP, and NO-E were involved in the conceptualization of the project and in the acquisition and analysis of the data. MD and JJ were involved in the interpretation of the data. All authors were involved in the drafting and revising of the work for intellectual content, provided approval for submission of the contents for publication, and agreed to be accountable for the accuracy and integrity of the project.

## Conflict of Interest

The authors declare that the research was conducted in the absence of any commercial or financial relationships that could be construed as a potential conflict of interest.
